# Minimal number of runs and the sequential scheme for local discrimination between special unitary operations

**DOI:** 10.1038/srep26696

**Published:** 2016-05-25

**Authors:** Tian-Qing Cao, Ying-Hui Yang, Zhi-Chao Zhang, Guo-Jing Tian, Fei Gao, Qiao-Yan Wen

**Affiliations:** 1 State Key Laboratory of Networking and Switching Technology, Beijing University of Posts and Telecommunications, Beijing, 100876, China; 2 School of Mathematics and Information Science, Henan Polytechnic University, Jiaozuo, 454000, China

## Abstract

It has been shown that any two different multipartite unitary operations are perfectly distinguishable by local operations and classical communication with a finite number of runs. Meanwhile, two open questions were left. One is how to determine the minimal number of runs needed for the local discrimination, and the other is whether a perfect local discrimination can be achieved by merely a sequential scheme. In this paper, we answer the two questions for some unitary operations *U*_1_ and *U*_2_ with 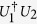 locally unitary equivalent to a diagonal unitary matrix in a product basis. Specifically, we give the minimal number of runs needed for the local discrimination, which is the same with that needed for the global discrimination. In this sense, the local operation works the same with the global one. Moreover, when adding the local property to *U*_1_ or *U*_2_, we present that the perfect local discrimination can be also realized by merely a sequential scheme with the minimal number of runs. Both results contribute to saving the resources used for the discrimination.

Quantum operations, which include unitary operations, quantum measurements, and quantum channels, are an important subject in the fields of quantum control and quantum information theory^1^,^2^,^3^. Recently, the discrimination of quantum operations^4^,^5^,^6^,^7^,^8^,^9^, especially the discrimination of unitary operations^10^,^11^,^12^,^13^,^14^,^15^,^16^,^17^,^18^,^19^,^20^, has received extensive attention. Indeed, the study itself of the distinguishability for unitary operations is a fundamental problem in quantum information theory, and after successfully discriminating unitary operations, we can further employ them to accomplish many other quantum information processing tasks. It should be noted that we only need to discuss the discrimination between two unitary operations, which is due to the fact that the discrimination of multiple unitary operations has been reduced to that of two unitary operations^11^.

Two different unitary operations are said to be perfectly distinguishable, if there exists at least an input state such that two corresponding output states, generated by the two unitary operations acting on the input state respectively, are orthogonal. That is to say, the orthogonality of the two output states implies the distinguishability of the two unitary operations. Thus, the issue of discrimination of unitary operations can be simplified to the study of orthogonality of the corresponding output states. Generally speaking, there are two kinds of distinguishing schemes, or to say, generating orthogonal output states schemes, *i.e.* the parallel scheme and the sequential scheme. Suppose *U*_1_ and *U*_2_ are two different unitary operations to be distinguished. In the parallel scheme, there exists a finite number *N* and an input state |*ψ*〉 such that two output states 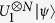 and 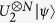 are orthogonal^13^. In the sequential scheme, there exist auxiliary unitary operations *X*_1_, ···, *X*_*N*_ and an input state |*ψ*〉 such that the output states *U*_1_*X*_*N*_*U*_1_ ···*X*_1_*U*_1_|*ψ*〉 and *U*_2_*X*_*N*_*U*_2_ ···*X*_1_*U*_2_|*ψ*〉 are orthogonal^13^. Furthermore, the minimal number of runs is the minimal number of times we apply the unknown unitary operation to make them perfectly distinguishable^11^.

In addition, the discrimination of unitary operations has been classified into two scenarios, *i.e.* the global one and the local one. In the global scenario, the unknown unitary operation is under the complete control of a single party who can perform any allowable physical operations^10^,^11^,^12^,^13^,^14^,^15^. It has been shown that any two different unitary operations can be perfectly distinguished, no matter by the parallel scheme^11^,^12^ or the sequential scheme^13^, when a finite number of runs are allowed. Besides, in both schemes, the minimal numbers of runs needed for the perfect discrimination are the same^11^,^13^. In the local scenario, which is the main point we shall discuss in this paper, the unknown unitary operation is shared by several physically distant parties. So local operations and classical communication (LOCC) are natural requirements for each party when they try to accomplish the discrimination. Such a restriction makes the discrimination of unitary operations more complicated^16^,^17^,^18^,^19^,^20^. Interestingly, Zhou *et al*.^16^ presented that any two different multipartite unitary operations are perfectly distinguished by LOCC with multiple runs. Later, Duan *et al*.^17^ independently proved the same result by introducing the theory of local numerical range. Specifically, they pointed out that any two different unitary operations can be locally distinguished by the parallel scheme or first sequential then parallel scheme after a finite amount of runs.

As is shown in ref. 13, the minimal number of runs can save the temporal resources, and the sequential scheme can save the spatial resources due to the fact that in the sequential scheme no entanglement or joint quantum operations are needed. So in order to save resources as much as possible, it is natural to ask the following two questions: What is the minimal number of runs needed for the above parallel scheme, even for any distinguishing scheme, no matter parallel or sequential? Can the perfect local discrimination be completed by merely a sequential scheme? Both questions, also referred to in ref.^17^, need further considerations. Yet until now there has been no relevant progress about the research, even for a special class of unitary operations.

In this paper we answer the two questions for some unitary operations. In detail, suppose *U*_1_ and *U*_2_ are any two different unitary operations on the *s* ⊗ *t*(*s, t* ≥ 2) quantum system such that 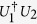 is local unitary equivalent to 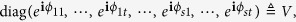 where *ϕ*_*ij*_ ∈ [0, 2*π*) and Θ(*V*) ∈ (0, *π*]. If the endpoints of Θ(*V*) are *ϕ*_*ij*_ and *ϕ*_*il*_, or *ϕ*_*ij*_ and *ϕ*_*hj*_, then the minimal number of runs needed for the local discrimination equals that needed for the global scenario, *i.e.* 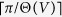, in which 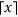 denotes the smallest integer that is not less than *x*, and Θ(*V*) represents the length of the smallest arc containing all the eigenvalues of *V* on the unit circle^11^. In this sense the local operation achieves the same function with the global one. Furthermore, when adding the local property to *U*_1_ or *U*_2_, by merely a sequential scheme the perfect local discrimination can be also accomplished with the minimal number of runs. Finally, the above results can be generalized to multipartite unitary operations.

## Results

### Local numerical range

We first introduce some definitions and results.

Consider a quantum system associated with a finite dimensional state space *H*. We denote the set of linear operations acting on *H* by *L*(*H*). In particular, *u*(*H*) is the set of unitary operations acting on *H*. Two unitary operations *U*_1_, *U*_2_ ∈ *u*(*H*) are said to be different if *U*_1_ is not the form *e*^**i***θ*^*U*_2_ for any real number *θ*. Let us introduce the notion of numerical range.

*Definition 1*. For *A* ∈ *L*(*H*), the numerical range of *A* is a subset of complex numbers defined as


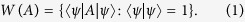


Suppose now we are concerned with a multipartite quantum system consisting of *m* parties, say, *M* = {*A*_1_, ···, *A*_*m*_}. Assume that the party *A*_*k*_ has a state space *H*_*k*_ with dimension *d*_*k*_. Then the whole state space is given by 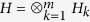 with total dimension *d* = *d*_1_ ···*d*_*m*_.

*Definition 2*^17^. *U* ∈ *u*(*H*) is said to be local or decomposable if 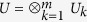 such that *U*_*k*_ ∈ *u*(*H*_*k*_). Otherwise *U* is nonlocal or entangled.

*Definition 3*^17^. The local numerical range of *A* is


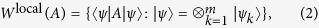


where |*ψ*_*k*_〉 ∈ *H*_*k*_ and 〈*ψ*_*k*_|*ψ*_*k*_〉 = 1.

Let *U* and *V* be two matrices on the *s* ⊗ *t* space. *U* and *V* are called local unitary equivalent if there exist *U*_1_ ∈ *u*(*s*) and *U*_2_ ∈ *u*(*t*) such that *U* = (*U*_1_ ⊗ *U*_2_)*V* (*U*_1_ ⊗ *U*_2_)^†^. Moreover, when *U* is local unitary equivalent to *V*, we can obtain that *U*^⊗*p*^ and *V*^⊗*p*^ are local unitary equivalent, and *W*^local^(*U*^⊗*p*^) = *W*^local^(*V*^⊗*p*^) for any *p* ∈ *N*^21^.

Next two relevant lemmas about the local discrimination of unitary operations will be presented.

*Lemma 1*^17^. Two different unitary operations *U*_1_ and *U*_2_ are perfectly distinguishable by LOCC in the single-run scenario if and only if 
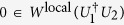
.

*Lemma 2*^17^. Suppose two different multipartite unitary operations *U*_1_ and *U*_2_ satisfy that 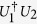 is non-Hermitian (up to some phase factor), then there exists a finite *N* such that 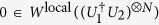.

Lemma 2 gives the existence of a finite number needed for the perfect discrimination in the local scenario.

For simplicity, in what follows we shall only consider the case in which *U*_1_ and *U*_2_ are both bipartite unitary operations acting on the *s* ⊗ *t*(*s, t* ≥ 2) space, and the multipartite case can be similarly discussed.

### Minimal number of runs for the local distinguishability

In this section, we mainly discuss the minimal number of runs needed for a perfect discrimination between two bipartite unitary operations in the LOCC scenario.

First, two different unitary operations *U*_1_ and *U*_2_ such that 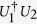 is local unitary equivalent to *V* are considered, where *V* is a diagonal unitary matrix in a product basis. According to that Θ(*U*) = Θ(*XUX*^†^) for any *X* ∈ *u*(*H*) and the local unitary transformations do not alter the product state nature of the basis in general, we have the theorem.

Theorem 1. *Let U*_1_ *and U*_2_ *be any two different bipartite unitary operations on the s* ⊗ *t*(*s, t* ≥ 2) *space such that* 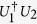 *is local unitary equivalent to*


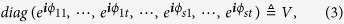


*where ϕ*_*ij*_ ∈ [0, 2*π*) *and* Θ(*V*) ∈ (0, *π*]*. If the endpoints of* Θ(*V*) *are ϕ*_*ij*_ *and ϕ*_*il*_*, or ϕ*_*ij*_ *and ϕ*_*hj*_*, then* 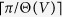 *is the minimal number of runs needed for distinguishing U*_*1*_ *and U*_*2*_ *locally with certainty.*

*Proof*. By the conditions, 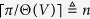 is the minimal number of runs needed for globally distinguishing *U*_1_ and *U*_2_ with certainty. Thus, if they are perfectly distinguished by LOCC, the minimal number of runs cannot be less than *n*. In the following we will illustrate that *n* is just the minimal number of runs by finding a parallel scheme to distinguish them locally.

Without loss of generality, suppose *U*_1_ and *U*_2_ are unitary operations consisting of two parties *A* and *B*, where *A* is *s*-dimensional, and *B* is *t*-dimensional. Let the endpoints of Θ(*V*) be *ϕ*_*ij*_ and *ϕ*_*il*_, where *j* < *l*.

In fact, we can find a bipartite product state


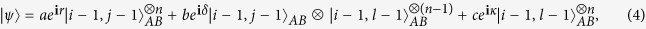


where *r, δ, κ* ∈ [0, 2*π*), and


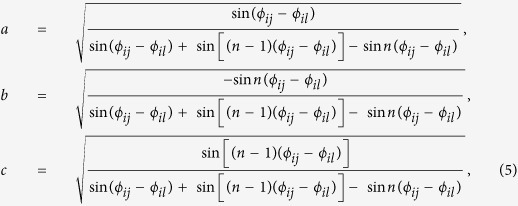


such that


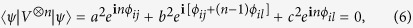


which means that when *U*_1_ and *U*_2_ are applied *n* times in parallel, they can be locally distinguished by Lemma 2.

To sum up, we can claim that 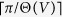 is the minimal number of runs needed for the perfect discrimination between *U*_1_ and *U*_2_ in the LOCC scenario.

From the above proof, it is clear that the minimal number of runs needed for the local discrimination is the same with that needed for the global scenario. The fact reveals a counterintuitive result: For the perfect discrimination of two unitary operations as in Theorem 1, the global operation has no advantages over the local one. As an illustrative example of Theorem 1, consider a special case where *U*_1_ and *U*_2_ are two 2 ⊗ 3 unitary operations satisfying that


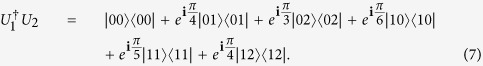


One can directly see that 
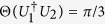
, and the endpoints of 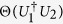 are *ϕ*_11_ = 0 and *ϕ*_13_ = *π*/3. As we can find a bipartite product state 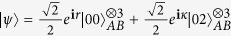, where *r, κ* ∈ [0, 2*π*), such that 
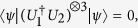
 then 
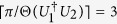
 is the minimal number of runs needed for the perfect discrimination of *U*_1_ and *U*_2_ in the LOCC scenario.

From Theorem 1, one can see that the endpoints of Θ(*V*) being *ϕ*_*ij*_ and *ϕ*_*il*_, or *ϕ*_*ij*_ and *ϕ*_*hj*_ are just the sufficient conditions. So when will they be also the necessary conditions? The following corollary will give an answer.

Corollary *Let U*_1_ *and U*_2_ *be any two different bipartite unitary operations on the s* ⊗ *t*(*s, t* ≥ 2) *space such that* 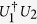 *is local unitary equivalent to*


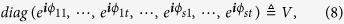


*where ϕ*_*ij*_ ∈ [0, 2*π*) *and* Θ(*V*) = *π. They are perfectly distinguished by LOCC in the single-run scenario if and only if the endpoints of* Θ(*V*) *are ϕ*_*ij*_ *and ϕ*_*il*_*, or ϕ*_*ij*_ *and ϕ*_*hj*_.

*Proof*. It suffices to show the necessity.

Without loss of generality, suppose the endpoints of Θ(*V*) are *ϕ*_*ij*_ and *ϕ*_*hl*_, where *i* < *h, j* ≠ *l* and *ϕ*_*ij*_ < *ϕ*_*hl*_. We have *ϕ*_*hl*_ = *ϕ*_*ij*_ + *π*.

According to that *U*_1_ and *U*_2_ are locally distinguished with a single run, there must be an *s* ⊗ *t* product state


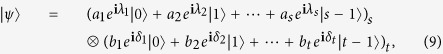


where *a*_*k*_ ≥ 0, *b*_*p*_ ≥ 0, 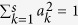, 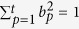, and *λ*_*k*_, *δ*_*p*_ ∈ [0, 2*π*), such that 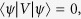 which is equivalent to


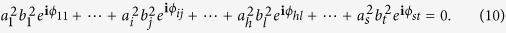


Let *ϕ*_*ij*_ = 0 and other *ϕ*_*mn*_ ∈ (0, *π*). We have *ϕ*_*hl*_ = *π*. Further 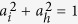, 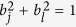, and 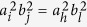. A routine calculation shows that *a*_*i*_ = *b*_*l*_ and *a*_*h*_ = *b*_*j*_. By *j* ≠ *l*, we can get 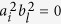 and 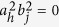. Thus, *b*_*j*_ = *b*_*l*_ = 0, which is a contradiction.

Corollary indicates that for any two different unitary operations constrained as above, our result is more practical and efficient than Lemma 1 in determining their local distinguishability in the single-run scenario. Because we do not need to compute the local numerical range which itself is generally difficult to calculate^22^. To see this, take *U*_1_ and *U*_2_ such that


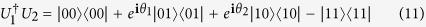


for 0 < *θ*_1_, *θ*_2_ < *π*. It is clear that 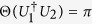, and the endpoints of 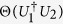 are *ϕ*_11_ = 0 and *ϕ*_22_ = *π*. By Corollary, we can immediately claim that *U*_1_ and *U*_2_ cannot be locally distinguished in the single-run scenario, without complicated calculations to demonstrate 
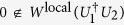
 as in ref. 17.

### Sequential scheme of the local distinguishability

In this part, we primarily focus on the question that whether a perfect local discrimination can be achieved by merely a sequential scheme. The solution to the question is helpful to save the spatial resources as no entanglement or joint quantum operations are needed in the sequential scheme. Fortunately, a positive answer will be made.

Theorem 2 *Let U*_1_ *and U*_2_ *be any two different s* ⊗ *t*(*s, t* ≥ 2) *unitary operations such that* 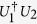 *is local unitary equivalent to*


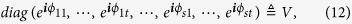


*where ϕ*_*ij*_ ∈ [0, 2*π*) *and* Θ(*V*) ∈ (0, *π*]*. Suppose one of them is local and* 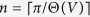
*. If the endpoints of* Θ(*V*) *are ϕ*_*ij*_ *and ϕ*_*il*_*, or ϕ*_*ij*_ *and ϕ*_*hj*_*, then there exist local unitary operations X*_1_, *X*_2_, ···, *X*_*n*−1_ *and an s* ⊗ *t product state* 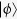 *such that*


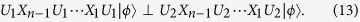


*Proof*. By Theorem 1, *n* is not only the minimal number of runs needed for a perfect local discrimination, but also can be achieved in the parallel scheme.

Here we will illustrate that when *U*_1_ and *U*_2_ are sequentially applied *n* runs, they can be locally distinguished. Therefore, the minimal number of runs *n* is also realized when *U*_1_ and *U*_2_ are perfectly distinguished by LOCC with the sequential scheme. The method in the proving process is inspired by Duan *et al*.^13^.

Without loss of generality, let


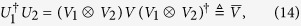


where *V*_1_ is an *s* × *s* unitary matrix and *V*_2_ is *t* × *t*. Suppose *U*_1_ is local, and the endpoints of Θ(*V*) are *ϕ*_*ij*_ and *ϕ*_*il*_, where *j* < *l*.

First, we consider the case *U*_1_ = *I* and 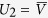. Subsequently the general case can be reduced to this special one.

Next we will prove that there always exists a local unitary operation 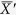 and an *s *⊗ *t* product state 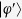 such that 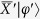 and 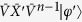 are orthogonal. In other words, through being sequentially applied *n* times, *I* and 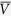 can be locally discriminated.

Let 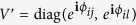. Suppose


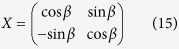


is the general form of real unitary matrices on the 2-dimensional space. We will find *β* satisfying tr(*X*^†^*V*′*XV*′^*n*−1^) = 0 as follows.

A routine calculation shows that


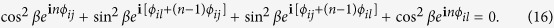


Combining with the sum-to-product identities and 
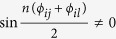
, or 
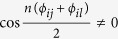
, we can get that


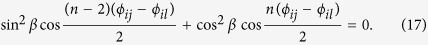


Further,


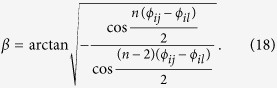


Thus, for above *β, X*^†^*V*′*XV*′^*n*−1^ has two opposite eigenvalues. Further we can assume the spectral decomposition as


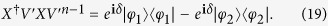


Let


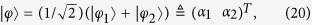


where *T* denotes the matrix transposition. Thus, we have found *β, X* and 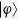 such that 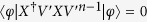.

Suppose


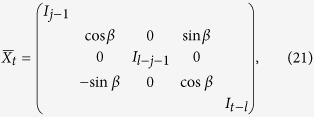



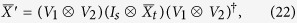


and


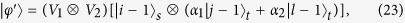


where *I*_*s*_ represents the *s* × *s* identity matrix. We can conclude that


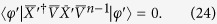


Second, for general *U*_1_ and *U*_2_ satisfying 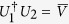, there always exists the local unitary operation 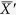 and the *s* ⊗ *t* product state 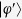 as above such that


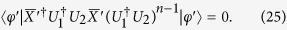


Finally, suppose 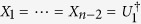, 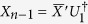, and 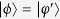 in the theorem. Then


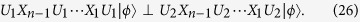


It can be seen from Theorem 2 that there indeed exist some unitary operations such that their local discrimination can be completed by merely a sequential scheme, and meanwhile we present an explicit protocol of the local discrimination without any entanglement or joint quantum operations. Interestingly, the minimal number of runs is also *n*, which is the same with that in the global scenario. All these make the local discrimination actually feasible in experiment.

As an application of Theorem 2, consider a particular case where *U*_1_ and *U*_2_ are two-qubit unitary operations satisfying that *U*_1_ is local and


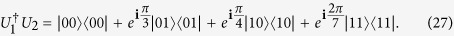


One can directly see that 
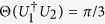
, *n* = 3, and the endpoints of 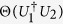 are *ϕ*_11_ = 0 and *ϕ*_12_ = *π*/3. Let 
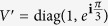
. We can find *β* = 0, *X* = *I*_2_, and 
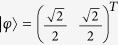
, where *T* denotes the matrix transposition, such that 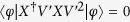. Suppose 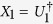, 
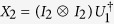
, and 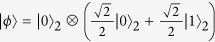, we have 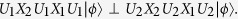 Therefore, it has been shown that the perfect local discrimination of *U*_1_ and *U*_2_ can be achieved by merely a sequential scheme.

## Discussion

The local discrimination of two unitary operations *U*_1_ and *U*_2_ discussed in refs 19 and 20 is in the single-shot scenario. Bae^19^ mainly investigated the relations between the discrimination and the entangling capabilities of given *U*_1_ and *U*_2_, and drew the conclusion that there exist non-entangling unitary operations being perfectly distinguishable only for global operations. While Cao *et al*.^20^ presented a necessary and sufficient condition, which can be employed to efficiently determine the perfect local distinguishability of *U*_1_ and *U*_2_ satisfying 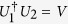 with *V* being a two-qubit diagonal unitary matrix.

Compared to the results in refs 19 and 20, our study on the local discrimination of unitary operations in this paper is primarily in the multiple-runs scenario. We have determined the minimal number of runs and put forward the sequential scheme for the local discrimination of some unitary operations. Concretely, for any two different unitary operations with certain limitations, we show that the minimal number of runs needed for the local discrimination is equal to that needed for the global scenario, which means that the local operation achieves the same function with the global one. Furthermore, when one more condition is restricted to the two unitary operations, we present that by merely a sequential scheme the perfect local discrimination can be also completed with the minimal number of runs. Both results are benefit for saving the resources, temporal or spatial, which are crucial in practice.

Despite the above research progress, we have yet neither determined the minimal number of runs, nor given the existence of an effective merely-sequential scheme for the local discrimination of two general unitary operations. But we believe that the results about the special unitary operations can provide new insight into the study of the two questions and help us to make a further research.

## Additional Information

**How to cite this article**: Cao, T.-Q. *et al*. Minimal number of runs and the sequential scheme for local discrimination between special unitary operations. *Sci. Rep.* **6**, 26696; doi: 10.1038/srep26696 (2016).
